# Inference of Relationships in Population Data Using Identity-by-Descent and Identity-by-State

**DOI:** 10.1371/journal.pgen.1002287

**Published:** 2011-09-22

**Authors:** Eric L. Stevens, Greg Heckenberg, Elisha D. O. Roberson, Joseph D. Baugher, Thomas J. Downey, Jonathan Pevsner

**Affiliations:** 1 Program in Human Genetics, Johns Hopkins School of Medicine, Baltimore, Maryland, United States of America; 2 Partek, St. Louis, Missouri, United States of America; 3 Program in Biochemistry, Cellular, and Molecular Biology, Johns Hopkins School of Medicine, Baltimore, Maryland, United States of America; 4 Department of Neurology, Hugo Moser Institute at the Kennedy Krieger Institute, Baltimore, Maryland, United States of America; 5 Department of Neuroscience, Johns Hopkins School of Medicine, Baltimore, Maryland, United States of America; University of Alabama at Birmingham, United States of America

## Abstract

It is an assumption of large, population-based datasets that samples are annotated accurately whether they correspond to known relationships or unrelated individuals. These annotations are key for a broad range of genetics applications. While many methods are available to assess relatedness that involve estimates of identity-by-descent (IBD) and/or identity-by-state (IBS) allele-sharing proportions, we developed a novel approach that estimates IBD0, 1, and 2 based on observed IBS within windows. When combined with genome-wide IBS information, it provides an intuitive and practical graphical approach with the capacity to analyze datasets with thousands of samples without prior information about relatedness between individuals or haplotypes. We applied the method to a commonly used Human Variation Panel consisting of 400 nominally unrelated individuals. Surprisingly, we identified identical, parent-child, and full-sibling relationships and reconstructed pedigrees. In two instances non-sibling pairs of individuals in these pedigrees had unexpected IBD2 levels, as well as multiple regions of homozygosity, implying inbreeding. This combined method allowed us to distinguish related individuals from those having atypical heterozygosity rates and determine which individuals were outliers with respect to their designated population. Additionally, it becomes increasingly difficult to identify distant relatedness using genome-wide IBS methods alone. However, our IBD method further identified distant relatedness between individuals within populations, supported by the presence of megabase-scale regions lacking IBS0 across individual chromosomes. We benchmarked our approach against the hidden Markov model of a leading software package (PLINK), showing improved calling of distantly related individuals, and we validated it using a known pedigree from a clinical study. The application of this approach could improve genome-wide association, linkage, heterozygosity, and other population genomics studies that rely on SNP genotype data.

## Author Summary

High-density microarrays measuring single nucleotide polymorphisms (SNPs) provide information about the genotypes across many loci. SNP genotypes observed for any two individuals can be compared in terms of identity-by-state (IBS), in which two individuals are observed to have 0, 1, or 2 alleles in common at a given locus, across a chromosomal region, or throughout the genome. These alleles may be shared identical-by-descent (IBD) in which 0, 1, or 2 alleles are inherited from a recent common ancestor, or they may be identical by chance because the allele is frequent in the population. The expected proportion of genome sharing between two individuals varies as a function of their genetic relatedness. We introduce a method to estimate IBD that can be used to analyze relatedness in pedigrees or in large-scale population studies with thousands of individuals. This can be combined with observed IBS to distinguish a variety of types of relatedness, providing theoretically justified results that are graphed in a manner that is straightforward to interpret. The methods we introduce are relevant to a variety of SNP applications including linkage and association studies and population genomics studies.

## Introduction

Single nucleotide polymorphism (SNP) genotyping is used to delineate the extent and nature of chromosomal variation, examine population genetic structure, and find loci that contribute to disease. SNPs are used as proxies for the unobserved sequence variants in the surrounding DNA, allowing measurement of the flow of genetic material through populations [1].

There are important limitations for using SNPs to identify causal disease variants. Genome-wide association studies (GWAS) rely on representative sampling of a subset of individuals from a population. Therefore, calculations testing the association between alleles, the frequency of alleles in the population, and the contribution of alleles to a phenotype must use estimates of the population allele frequency based on the representative sampling. These estimates of allele frequencies are sensitive to inflation or deflation when the genotyping data are derived from individuals with unreported familial relationships or with admixed ancestry (potentially leading to population stratification).

For any given pair of individuals with genotype information, identity-by-state (IBS) can be observed at a given locus with three possible outcomes: the individuals have two different alleles (IBS0) or they share one (IBS1) or two (IBS2) alleles in common. For example, a pair of individuals with genotypes AA and BB are IBS0 at this locus whereas a pair with AA and AB are IBS1. Two individuals who share 1 or 2 alleles IBS at a given locus may have inherited the shared allele(s) from a recent common ancestor, in which these allele(s) are identical-by-descent (IBD). IBD approaches have been applied to linkage mapping [2] in which segments of IBD are detected with informative SNPs. IBD regions tend to be small between pairs of individuals derived from a given population that are not closely related, primarily because their last common ancestor was many generations ago. As such, GWAS are predicated upon the detection of regions of IBD when stratifying by phenotype.

In this study we demonstrate an approach that combines our IBD method with IBS information to estimate the relatedness between individuals in pedigrees and/or in large population-based studies. There are two main aspects of this work. First, we introduce plots based in part on methods suggested by Lee [3] and Rosenberg [4] to analyze a subset of informative IBS observations. We implement Lee's mathematical approach to characterizing genetic relatedness based on the ratio of concordant heterozygotes (i.e. AB/AB genotype calls) divided by the sum of concordant heterozygotes plus discordant homozygotes (e.g. AB/AB plus AA/BB) [3]. This metric represents the x-axis in several of our figures below. Additionally, we use IBS as the basis for a metric that graphically distinguishes relatedness consistent with earlier work [4]. Second, we introduce a method to calculate IBD in comparisons between two individuals, providing highly accurate estimates of Cotterman coefficients of relatedness (K0, K1, and K2 are our estimates of Cotterman coefficients k0, k1, k2) [5].

Combining these IBS and IBD approaches, our analyses simultaneously reveal previously unknown familial relationships and population substructure in large-scale SNP data. Notably, our method applies to pedigrees but does not rely on prior knowledge of relationships or ethnicity. While other exploratory techniques such as principal components analysis (PCA) of genotype data can indicate outliers, the nature of such relationships is not explicitly described. In contrast, our method is useful to define relationships. We observed differences within and between populations (and pedigrees) due to multiple factors including familial relationships, autosomal heterozygosity rate, chromosomal anomalies, and population admixture. We analyzed data from the Coriell Institute's National Institute of General Medical Sciences (NIGMS) Human Genetic Cell Repository Human Variation Panel (referred to as the Human Variation Panel), an extensively used data source, and found undocumented familial relationships.

Our IBD method uses an overlapping window approach (see Methods) and is comparable to that of PLINK [6], which employs a hidden Markov model to infer underlying IBD in chromosomal segments based on observed IBS states. Similar to PLINK's HMM, we analyze SNPs in a genome-wide fashion to detect patterns of IBS0, IBS1, and a subset of IBS2, and further infer regions of IBD sharing that are estimates of Cotterman coefficients of relatedness k0, k1, and k2. These IBD estimates are not reliant on prior sample annotation or haplotype data. Our approach, however, reports fewer false positives (defined as individuals who are unrelated based on IBS sharing, but who are called as related) relative to PLINK's HMM. Other methods for inferring IBD relatedness using SNP data include GERMLINE, BEAGLE IBD, and fastIBD [7], [8], [9]. These are based on identifying shared haplotypes and rely on haplotype maps of the human genome [10], [11], [12], [13]. These programs allow for a robust detection of shared haplotypes for regions as small as 2cM (∼2 Mb). Other methods for estimating kinship coefficients, paternity indices and other relationship indices in the forensic and genetic literature do not rely on haplotype data. For example, EMMAX (efficient mixed-model association eXpedited) addresses kinship and population stratification using a variance components approach [14]. Related individuals that share, on average, longer stretches of IBD (10 Mb for example) are identified with very high confidence levels [8]. The approach we introduce is robust in detecting shared segments between individuals of recent ancestry. It provides accurate IBD estimates allowing for improved inference of relationships.

## Results

### Unexpected relationships among individuals self-declared as unrelated in the Human Variation Panel

The Human Variation Panel consists of four populations (individuals of African-American ancestry [AA], Caucasian ancestry [CAU], Han Chinese ancestry [CHI], and Mexican-American ancestry [MEX]; n = 100 per group). All samples from these individuals were submitted to the NIGMS repository with annotation indicating they were unrelated. Using autosomal SNP genotype data (n = 872,242 SNPs) from these samples, we analyzed all pairwise IBS relationships in each population group (n = 19,800 comparisons).

**Figure 1:**
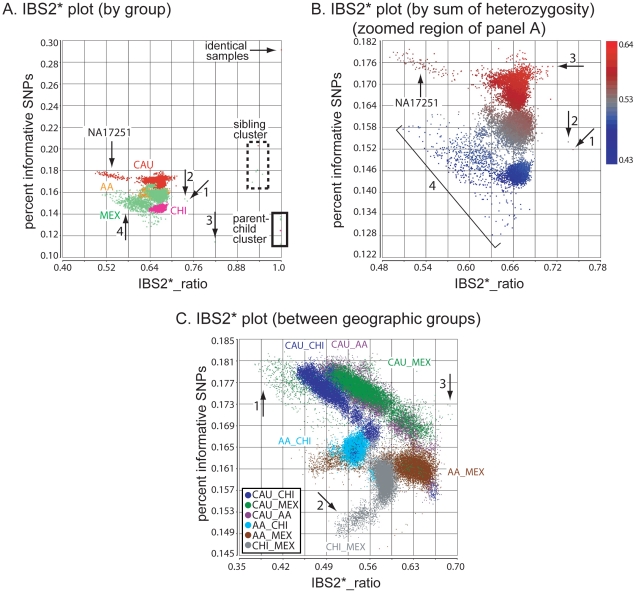
Genetic relatedness plots of the Human Variation Panel genotype data. Abbreviations: AA, African American; CAU, Caucasian; CHI, Chinese; MEX, Mexican. (A) IBS2* plot of the within-group comparisons (n = 19,800). The IBS2*_ratio values are centered on 2/3 for unrelated individuals within a population. The relationship of NA17251 to 99 other AA individuals is indicated (arrow). A group of 9 MEX individuals have atypically low heterozygosity rates and form a cluster separated from other within-MEX comparisons (arrow 1). (B) IBS2* plot in which pairwise comparisons with IBS2*_ratio values >0.8 are removed (n = 13) and data points are colored by the sum of autosomal heterozygosity of each pair of individuals. (C) IBS2* plot for between-group comparisons (n = 60,000) for which none are expected to be genetically related. For groups having individuals with large differences in heterozygosity rates, such as AA-CHI comparisons, the IBS2*_ratio values are significantly lower than 2/3. The MEX individuals with atypical heterozygosity rates tend to form outlier clusters in between-group comparisons such as AA-MEX (arrow 1) and CHI-MEX (arrow 2). A group of five pairwise comparisons having relatively high IBS2*_ratio values (0.685 to 0.692; arrow 3) involve MEX individual NA17709 in comparison to CAU individuals.

For the four within-population comparisons we generated a plot (referred to as an IBS2* plot) having x-axis values referred to as IBS2*_ratio and based on the ratio of IBS2*/(IBS0 + IBS2*), suggested by Lee [3]. We plotted y-axis values termed percent informative SNPs and consisting of the sum of (IBS0 + IBS2*) divided by all IBS counts (IBS0+IBS1+IBS2; Figure 1A). For each population we observed a major cluster of data points having IBS2*_ratio values near 0.66–0.67; these values were expected to form a normal distribution centered at 2/3 for unrelated individuals (see Methods). We implemented a two-sided statistical test of the null hypothesis that a given pairwise comparison does not have an IBS2*_ratio value either significantly >2/3 (indicating familial relatedness) or <2/3 (indicating different allele frequencies between the compared samples accounted for by phenomena such as population admixture or reduced heterozygosity due to stretches of homozygosity [lacking AB calls]; see Methods).

We used a Z-test (as suggested by Lee [3]), and measured the p-value for every pairwise comparison. We observed that p-values ≤0.000025 (including a Bonferroni correction for 19,800 tests) were found in comparisons greater than 0.672 and less than 0.661 suggesting a very narrow range of IBS2*_ratio values for which the null hypothesis was not rejected. We note that an IBS2*_ratio value greater than 0.70 was used empirically to highlight potential pairwise comparisons suspected to be related.

**Figure 2:**
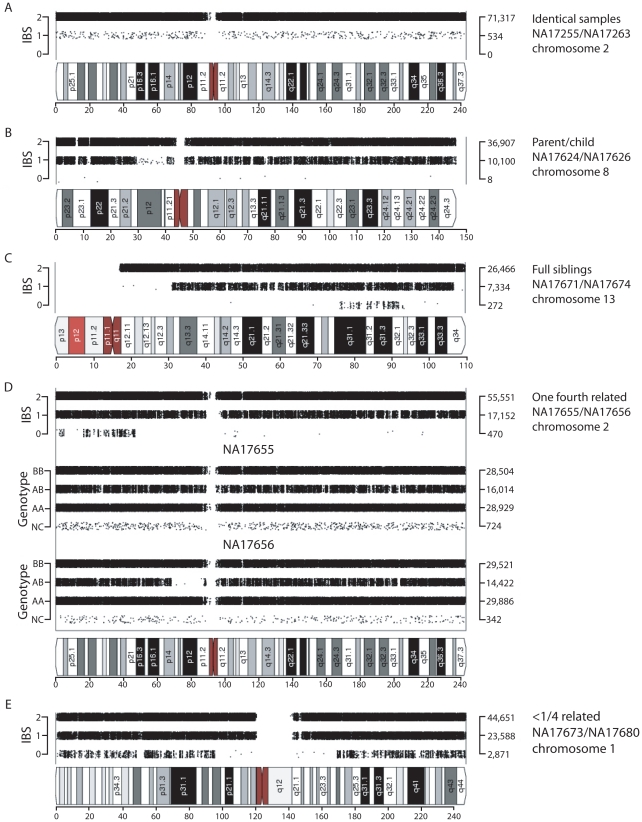
Visualization of shared chromosomal regions based on IBS for related individuals. IBS values for comparisons of two individuals are shown for a representative chromosome for the following pairs: (A) replicate samples NA17255/NA17263, (B) parent-child NA17624/NA17626, (C) full siblings NA17671/NA17674, (D) individuals sharing one quarter of their alleles (NA17655/NA17656; e.g. half-siblings), (E) distantly related individuals NA17673/NA17680. Data analysis was performed using SNPduo software. Note that for pericentromeric regions and the short arms of acrocentric chromosomes (as in panel C) no SNP data were available, producing no IBS measurements.

Given that the Human Variation Panel had no previously annotated familial relationships or replicate samples, we expected no IBS2*_ratio values >2/3 (e.g. 0.70). Surprisingly, we observed 25 data points with values >0.70 that potentially corresponded to familial relationships (Table 1 includes a subset of 16 of these pairwise comparisons for which we obtained evidence of familial relationships, as discussed below; 6 other relationships in the table with IBS2*_ratio values <0.70 are described below). The CAU group included a pair of identical samples (Figure 1A arrow, corresponding to NA17255/NA17263). The IBS2*_ratio value was near 1.0 for this pairwise comparison, as expected for identical samples that lack essentially all IBS0 calls. This relationship is supported by plotting IBS for each chromosomal position across all autosomes using SNPduo software [15], a program that performs pairwise comparisons of SNP genotype data and plots IBS (as well as genotypes) for one chromosome or the entire genome. This revealed a predominant pattern of IBS2 as shown for chromosome 2 (Figure 2A). Typical of other genetically identical samples analyzed with low genotyping error rates, these two individuals shared only 11 IBS0 calls and 6,410 IBS1 calls in contrast to 838,898 IBS2 calls from autosomal loci. The samples were annotated by the Human Genetic Cell Repository as a 6 year-old boy (NA17255) and a 26 year-old female (NA17263). The two samples were likely to be technical replicates for the 6 year-old boy based on a lack of AB calls on the X chromosome (data not shown).

**Table 1:** Unexpected relationships inferred in the Coriell Human Variation Panel.

IID1	IID2	IBS2*_ratio	Group	K0	K1	K2	Z0*	Z1*	Z2*	Z0	Z1	Z2	Rel
NA17255	NA17263	1.000	CAU	0.000	0.000	1.000	na	na	na	0.000	0.025	0.975	ID
NA17837	NA17846	0.998	CHI	0.000	0.999	0.001	0.004	0.996	0.000	0.004	0.996	0.000	PC
NA17831	NA17846	0.998	CHI	0.000	0.999	0.001	0.006	0.993	0.002	0.006	0.992	0.002	PC
NA17686	NA17687	0.998	MEX	0.000	0.999	0.001	na	na	na	0.004	0.972	0.024	PC
NA17629	NA17630	0.998	MEX	0.000	0.997	0.003	0.004	0.996	0.000	0.004	0.996	0.000	PC
NA17644	NA17687	0.999	MEX	0.000	0.997	0.003	na	na	na	0.003	0.974	0.023	PC
NA17624	NA17626	0.998	MEX	0.000	0.991	0.009	na	na	na	0.003	0.836	0.161	PC
NA17671	NA17674	0.921	MEX	0.243	0.567	0.190	0.256	0.536	0.209	0.256	0.536	0.208	FS
NA17644	NA17686	0.949	MEX	0.197	0.525	0.278	0.172	0.504	0.324	0.172	0.505	0.324	FS
NA17672	NA17674	0.931	MEX	0.233	0.517	0.251	0.235	0.500	0.265	0.235	0.501	0.264	FS
NA17655	NA17656	0.818	MEX	0.499	0.499	0.002	0.397	0.420	0.183	0.397	0.420	0.183	2°
NA17671	NA17672	0.935	MEX	0.236	0.496	0.268	0.226	0.502	0.272	0.226	0.503	0.271	FS
NA17294	NA17295	0.937	CAU	0.258	0.471	0.271	0.229	0.514	0.257	0.229	0.515	0.257	FS
NA17673	NA17680	0.742	MEX	0.766	0.234	0.000	0.731	0.269	0.000	0.731	0.269	0.000	3°
NA17454	NA17459	0.735	MEX	0.800	0.200	0.001	0.778	0.209	0.013	0.779	0.209	0.012	3°
NA17203	NA17257	0.711	CAU	0.866	0.134	0.000	0.853	0.140	0.007	0.854	0.140	0.006	<3°
NA17289	NA17299	0.671	CAU	0.899	0.101	0.000	0.986	0.000	0.014	0.986	0.000	0.014	<3°
NA17785	NA17794	0.673	CHI	0.941	0.058	0.001	0.985	0.003	0.012	0.985	0.002	0.013	<3°
NA17626	NA17655	0.682	MEX	0.955	0.045	0.000	na	na	na	0.816	0.000	0.184	<3°
NA17626	NA17656	0.652	MEX	0.964	0.036	0.000	na	na	na	0.820	0.000	0.180	<3°
NA17615	NA17702	0.675	MEX	0.965	0.035	0.000	0.978	0.022	0.000	0.978	0.023	0.000	<3°
NA17632	NA17695	0.689	MEX	0.968	0.032	0.000	0.920	0.080	0.000	0.920	0.080	0.000	<3°

Abbreviations: IID1 and IID2, individual identifiers 1 and 2; Group, Caucasian (CAU), Chinese (CHI), or Mexican (MEX); PC, parent-child; FS, full sibling; ID, identical; K0, K1, K2 estimates of IBD0, IBD1, IBD2, respectively; Z0*, Z1*, Z2*, IBD estimates based on PLINK's HMM with individuals with low genotyping rates removed; Z0, Z1, Z2, IBD estimates based on PLINK's HMM with all individuals; Rel, estimation of relationship inferred from K1 and K2 values;

2°, second-degree relationship (e.g. avuncular);

3°, second-degree relationship (e.g. first-cousin). Entries are given in order of most to least K1, but with identical samples given first.

Putative parent-child relationships (Figure 1A) also had IBS2*_ratio values near 1.0 (n = 6, IBS2*_ratio value range 0.998–0.999) with essentially no IBS0 observations as expected for annotated parent-child relationships. In contrast to replicate samples, parent-child relationships were also characterized by extensive IBS1 sharing (Figure 2B). We note that X chromosome SNPs were excluded for all comparisons because parent-child relationships involving father and son, having hemizygous genotypes interpreted as biallelic AA or BB calls, result in IBS0 that skew those IBS2*_ratio values lower to ∼0.95–0.97. The Y chromosome and mitochondrial SNPs were also excluded.

The y-axis (percent informative SNPs) of the IBS2* plot (Figure 1A) provided a useful separation of replicate samples from parent-child samples (both of which have IBS2*_ratios near 1 because they lack IBS0 calls). Since replicates have mostly IBS2 calls (including IBS2*), the percent informative SNPs for these samples was extremely close to their averaged heterozygosity rate. This distinguishes identical samples from parent-child pairs: for identical samples, every genotype comparison aligns to itself, and the equation for the percent informative SNPs reduces to IBS2* divided by the total number of SNPs. IBS2* reflects the number of AB calls in that sample that aligned with other AB's since IBS1 is unexpected (i.e. heterozygosity rate with variation due to genotype errors).

Inferred sibling comparisons (n = 5) were evident with IBS2*_ratio values ranging from 0.92 to 0.95. We defined this group (boxed in Figure 1A) as siblings because data points for all annotated sibling relationships from other datasets were located there. For all potential sibling pairs that we identified there were typical patterns of allele sharing with (1) blocks of IBS0, IBS1, and IBS2 that indicated unshared regions of IBD0, (2) blocks of IBS1 and IBS2 that indicated shared regions of IBD1, and (3) blocks of IBS2 that indicated IBD2 sharing. An example is shown in Figure 2C. A presumptive second-degree relationship had an IBS2*_ratio value of 0.82 (Figure 1A, arrow 3; Figure 2D), separable from potential third-degree relationships (Figure 1A, arrows 1–2; Figure 2E) and unrelated individuals.

IBS2* plot x-axis (IBS2*_ratio) values less than 2/3 reflected differences in heterozygosity values in pairwise comparisons either within or between geographic groups. For example, CAU individual NA17251 in comparison with other CAU individuals had a sum of heterozygosity of 56.7 +/- 0.01% in contrast to other CAU comparisons having values of 59.7 +/- 0.1% (Figure 1B, arrow). Among the MEX population, 9 individuals had pairwise heterozygosity sums that were outliers (Figure 1B, region 4). These low values were due to extended regions of homozygosity in these individuals (e.g. Figure 2D; see genotype calls of NA17656) and will be discussed in detail below. Regions of homozygosity in one (or both) individuals decreased the amount of IBS2* calls (i.e. instead of potential AB/AB observations, we instead observed AB/AA or AB/BB) and increased the amount of IBS0 calls (e.g. instead of potential AB/AA observations, we instead observed AA/AA or BB/AA), thus reducing the IBS2*_ratio value.

For 9 of the 25 comparisons with IBS2*_ratio values greater than 0.70 (but less than 0.714), chromosomal IBS analysis using SNPduo failed to reveal any regions lacking IBS0 calls that would imply the presence of IBD1. Additionally, these samples represented all CAU within-group comparisons and had among the highest pairwise sums of heterozygosity rates (Figure 1B, arrow 3). An increasing heterozygosity rate provides more opportunities for an AB genotype in one individual to align with an AB in a second individual to produce higher IBS2* levels and decreased IBS0 levels (resulting in a higher IBS2*_ratio). This led us to refine the interpretation of the alternate hypothesis of the IBS statistic: values above 2/3 (e.g. 0.70) are attributable either to relatedness for any level of heterozygosity or high heterozygosity rates in one or both individuals relative to their population levels (Figure 1B). These IBS2*_ratio values were consistent with those of distantly related individuals. We have observed empirically that these values may mimic relationships between individuals up to and including first cousins (or similar 1/8th relationships).

Comparisons between individuals from different Human Variation Panel geographic groups were all expected to represent pairs of unrelated individuals having IBS2*_ratio measurements <2/3. Consistent with this expectation, there were no IBS2*_ratio values >0.69 (n = 60,000 pairwise comparisons; Figure 1C). Pairwise comparisons that centered around 2/3 (or that were slightly greater) were inferred to have similar allele frequencies (e.g. MEX and CAU) or had one or both members of the pair with high heterozygosity rates (data not shown). This similarity could also reflect more recent shared ancestry than other between-group comparisons. The five data points with the highest IBS2*_ratio values (0.685 to 0.692; Figure 1C, arrow 3) corresponded to pairwise comparisons between individuals with the highest summed heterozygosity rates (NA17709 compared to NA17275, NA17283, NA17294, NA17295, and NA17298; each pair had >61% summed heterozygosity).

### Combined identity-by-state and identity-by-descent analysis: comparison to PLINK

**Figure 3:**
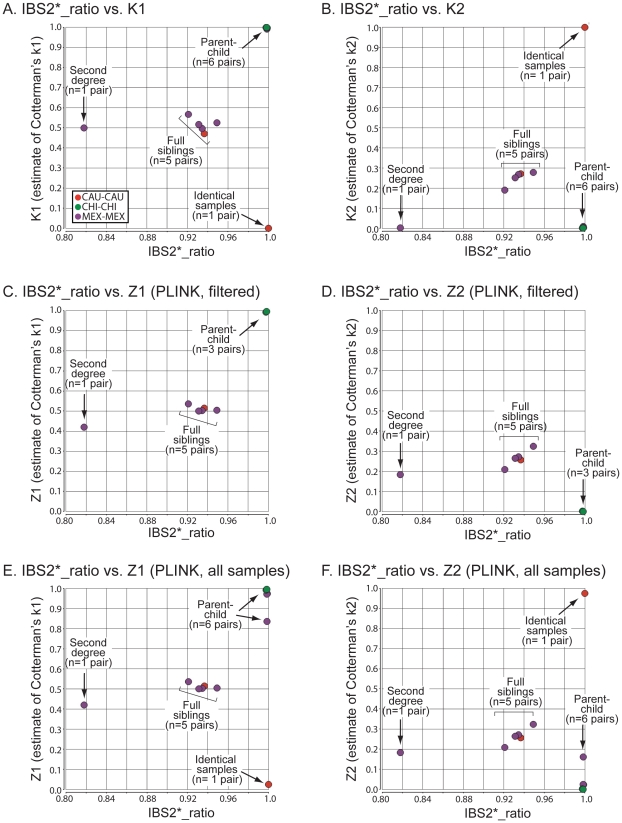
Relationship of IBS2* values to IBD estimates for recently related within-group comparisons. IBS2* plots for recently related within-group comparisons having IBS2*_ratio values >0.80. The y-axis shows IBD1 and IBD2 estimates derived from our approach (K1, panel A; K2, panel B), PLINK's HMM which had removed individuals due to low genotyping rates (Z1, panel C; Z2, panel D), and PLINK's HMM with the same quality control metrics except that no individuals were removed (Z1, panel E; Z2, panel F). Note that the x-axis and y-axis scales are the same for panels A–F. Arrows and brackets indicate groups of pairwise comparisons representing one relationship type (see text for details). Colors correspond to ethnic group and are matched across the four panels.

**Figure 4:**
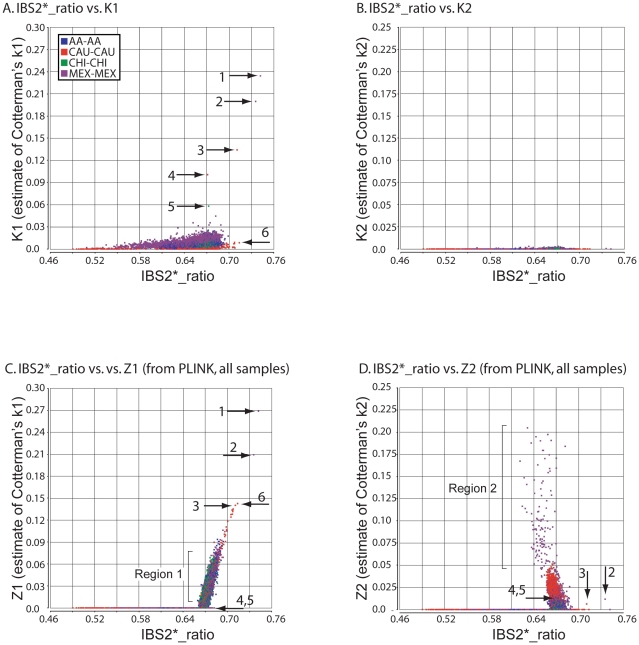
Relationship of IBS2* values to IBD estimates for distantly related within-group comparisons. IBS2* plots for within-group comparisons having IBS2*_ratio values <0.76 are shown. The y-axis shows IBD1 and IBD2 estimates derived from our approach (panels A, B) and PLINK's HMM which had removed individuals due to low genotype rates (panels C, D). Note that the x-axes scales are the same for all panels, and the y-axes are comparable for panels A, C and B, D. Arrows indicate pairwise comparisons: arrow 1, NA17673/NA17680 (MEX; see also arrow 1 in Figure 1A, 1B); arrow 2, NA17454/NA17459 (MEX; see also arrow 1 in Figure 1A, 1B); arrow 3, NA17203/NA17257 (CAU); arrow 4, NA17289/NA17299 (CAU); arrow 5, NA17785/NA17794 (CHI).

IBS can be used to infer IBD, which is further useful in defining relationships between individuals. PLINK software incorporates a method of moments approach using a hidden Markov model (HMM) to infer IBD from IBS data [6]. We developed an alternative approach (see Methods) to define IBD. We generated IBS2* plots of the Human Variation Panel within-group dataset in which the y-axis included IBD0, 1 or 2 estimates of Cotterman coefficients of relatedness k0, k1, k2. These were generated by our method (K0, K1, K2) or PLINK's HMM (Z0, Z1, Z2 using the notation provided by Purcell et al. [6]). We used PLINK to measure Z0, Z1, and Z2 using standard quality control measures in two different ways (see Methods) that resulted in the removal of 22 out of 400 samples (Table S1) in the first analysis but kept all of the samples for the second one. We divided our analysis into two sections. The first dealt with recently related individuals (i.e. those that were 1/4th related or more) and had an IBS2*_ratio >0.80 (Figure 3A–3F). The second focused on the remaining pairwise comparisons that had IBS2*_ratio values <0.76 (Figure 4A–4D).

For the 13 relationships that were previously inferred to be second-degree related or more based on the IBS2*_ratio, our method revealed expected estimates of Cotterman coefficients of relationship. For identical samples that are expected to have a K2 of 1.0, our method estimated zero IBD1 (Figure 3A; see arrow) and 100% IBD2 sharing (Figure 3B; see arrow). Several samples were removed by quality control procedures (see Methods) so that the first analysis by PLINK's HMM could not assign a Z1 or a Z2 estimate (Figure 3C, 3D): one of the identical samples (NA17255), NA17626 (putative parent-child relationship with NA17624), and NA17687 (putative parent-child relationship with inferred siblings NA17686 and NA17644). In the second analysis, in which no samples were removed, PLINK's HMM gave Z1 estimates of ∼0.02 (Figure 3E; see arrow) and Z2 estimates of ∼0.98 (Figure 3F; see arrow) for the identical samples. Both K1 and Z1 IBD1 estimates for putative parent-child relationships were 1.0, as expected (Figure 3A, 3C; see arrow). Notably, the Z1 given by PLINK's HMM was not at 1.0 for the parent-child relationships that were affected by the inclusion of low genotyping rate in one individual with Z1 estimates below 1 (Figure 3E) and Z2 estimates as high as 0.16 (Figure 3F).

IBD1 estimates (K1, Figure 3A; Z1, Figure 3C, 3E) were comparable for full sibling relationships which have an expected IBD1 coefficient of 0.5. Also, IBD2 estimates for our method (Figure 3B) and PLINK's HMM (Figure 3D, 3F) were centered on the expected coefficient of 0.25. PLINK's HMM estimated the putative second-degree relationship (NA17655/NA17656) as having a Z2 value of ∼ 0.18 (Figure 3D, 3F; see arrow) with an IBD1 estimate of 0.42 (Figure 3C, 3E; see arrow). In contrast we estimated the second-degree relationship to have a K1 of 0.50 (Figure 3A; see arrow), consistent with an expected value for putative second degree relatives, and a K2 of 0.002 (Figure 3B; see arrow), which was slightly higher than the expected coefficient of zero. SNPduo analysis provided evidence for IBD2 presence in the putative second-degree relationship with 10Mb on chromosome 10 (data not shown, but similar to the amount of IBD2 shown in Figure 2B for a putative parent-child relationship). However, IBS analysis using SNPduo did not indicate any other IBD2 sharing that would explain the high Z2 estimate of 0.18. This Z2 level approached the expected coefficient of IBD2 for siblings. We speculate that such a Z2 value could have been mistakenly interpreted as being associated with a full sibling relationship based on PLINK analysis alone.

For the great majority of pairwise comparisons, for which IBS2*_ratio values were centered on 2/3, we expected to observe IBD0 but little (if any) IBD1 or IBD2. Our method revealed extensive IBD0 (as measured by K0; data not shown) and very limited amounts of IBD1 and IBD2, as expected for unrelated individuals (Figure 4A, 4B). Based on IBS and IBD analyses, distantly related samples included MEX pairs NA17673/NA17680 and NA17454/NA17459 (Figure 4A; arrows 1 and 2); CAU pairs NA17203/NA17257 and NA17299/NA17289 (arrows 3 and 4); and CHI pairs NA17785/NA17794 (arrow 5). These five relationships were supported by visual inspection of chromosomal IBS (e.g. NA17673/NA17680 comparison in Figure 2E).

In our initial analyses using IBS measurements (Figure 1A), 25 comparisons had IBS2*_ratio values greater than 0.70, suggesting genetic relatedness. For 16 comparisons we independently inferred relatedness with K1 values from our IBD test. For nine comparisons that had IBS2*_ratio values greater than 0.70 but were due to high heterozygosity, our IBD test did not indicate K1 sharing (Figure 4A, arrow 6 represents an example in CAU individuals NA17275/NA17296; also see Figure 1B, arrow 3 showing the high heterozygosity of this pair). Thus, the addition of K1 information supported a model in which elevated IBS2*_ratio values may be attributed to atypical heterozygosity rather than familial relatedness.

PLINK's HMM reported dramatically more IBD1 for comparisons having IBS2*_ratio values approaching 2/3 and above (Figure 4C; see region 1). This could be because PLINK's HMM analyzes whole-genome IBS when calculating IBD probabilities [6]. In contrast, our method reports more samples with low K1 levels (up to 0.03) presumably due to our windowed approach. Notably, PLINK's HMM estimates of IBD1 were comparable to ours for several pairwise comparisons having elevated K1 values (Figure 4A, 4C arrows 1–3), but did not provide IBD1 estimates for several other comparisons (Figure 4A, 4C arrows 4 and 5). Also, a Z1 of ∼0.14 was assigned to a pair of individuals (NA17275/NA17296; Figure 4C, arrow 6) for whom our K1 estimate was very low (∼0.01; Figure 4A, arrow 6). We note that this corresponds to one of the CAU comparisons that had high heterozygosity, giving it a high IBS2*_ratio value. Thus we infer that the PLINK Z1 result was a false positive.

Our method revealed little IBD2 (Figure 4B) as expected for comparisons involving few related individuals. However, PLINK's HMM reported a large set of high IBD2 estimates centered at 2/3 (Figure 4D). Among the comparisons with the highest IBD2 estimates with Z2 values from ∼0.05 to 0.2 were those MEX outliers that had low heterozygosity due to contiguous regions of homozygosity (Figure 4D region 2). Since visual analysis using SNPduo did not provide evidence for relatedness such as the occurrence of SNPduo blocks lacking IBS0 or lacking IBS0 and IBS1 (indicating IBD1 and IBD2, respectively; data not shown), it is likely that most of these samples with high Z1 and Z2 estimates represented false positives. We note that IBD estimates provided by PLINK's HMM analysis with all samples included were almost identical to those provided when samples that had low genotyping call rates were removed. Therefore, we only present Z1 and Z2 estimates by PLINK's HMM analysis that included all samples.

To further characterize IBD relationships, we calculated IBS2* and IBD metrics for the Human Variation Panel between-group comparisons, which serve as a gold standard for authentically unrelated individuals. These plots again indicated very high levels of K0 (as expected; data not shown). The K1 and K2 estimates were very low (data not shown). PLINK is not designed for the analysis of IBD involving members of different populations [6], [16].

The IBD method we introduce lacked apparent false positive results that occurred using PLINK's HMM for the determination of distant genetic relatedness. The 21 comparisons (not including identical samples) that we identified as related were among those having the 22 highest K1 values. The most distantly related pair that we present as related (NA17632/NA17695) had a K1 value of 0.0323, while an unrelated pair had a comparable K1 value (0.0326). This is slighter higher than a theoretical 1/64th relationship (with a K1 of 0.03125) that appears to be the limit of detecting relatedness inferred by our IBD method. Some comparisons lacked an IBS2*_ratio greater than 0.70.

Based on our IBS and IBD analyses we identified previously unannotated familial relationships (Table 1) and reconstructed 14 pedigrees (Figure S1). The CAU group included a pair of identical samples, a sibling pair, and two distant relatives (<3rd degree). The CHI group included a father/mother/son trio and a pair of distant relatives (<3rd degree). In the MEX group we identified a mother and her two children; four siblings; two mother/daughter pairs; a 2nd degree relationship; two 3rd degree relationships; and three distant relationships (<3rd degree). After our analyses revealed unexpected familial relationships, Coriell provided nine additional samples for consideration as substitutes. None of these were closely related to each other or to any samples in the original panel (data not shown).

### Validation of familial assignments based on IBS2* plots

**Figure 5:**
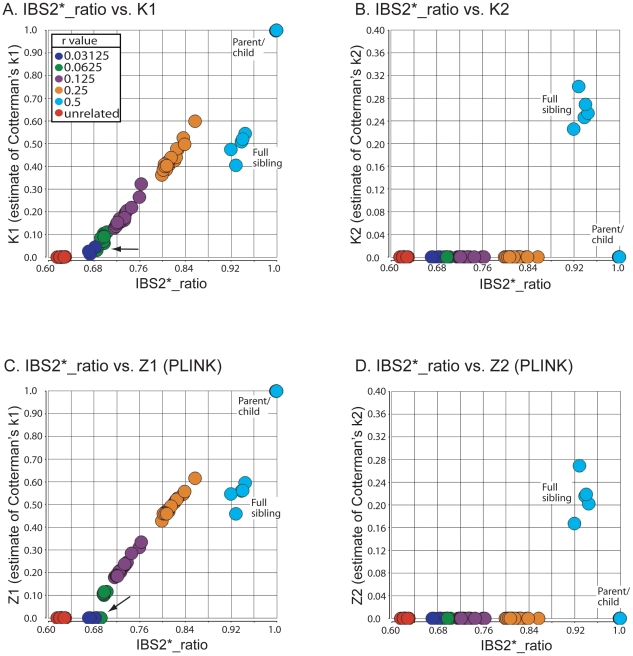
Validation of IBS2* methodology using annotated relationships from a known pedigree. IBS2* plot analysis of real data from a large pedigree annotated by distance (i.e. proportion of IBD). The y-axis shows IBD1 and IBD2 estimates derived from our approach (panels A, C) and PLINK's HMM (panels B, D). These distances refer to Cotterman coefficients of relatedness. Note the linear relationship between K1and Z1 and IBS2*_ratio values in panels A and C.

We validated the combined IBS and IBD approach by analyzing data from individuals with known familial relationships and compared it to PLINK's HMM. We used genotype data from a pedigree in a study of metachondromatosis [17]. This pedigree (see Figure S2) included known relationships from parent-child to first cousins that were twice-removed (e.g. a grandchild of individual X that is compared to individual X's first cousin). We analyzed IBS and IBD values in pairwise relationships (Table 2), and generated plots annotated by proportion IBD1 (K1, Figure 5A; Z1, Figure 5C). K1 and Z1 values increased comparably as a function of an increasing IBS2*_ratio. This was consistent with a decrease in IBS0 calls and an increase in the percent of the genome that was shared. Our K1 estimate was comparable to the Z1 estimate of PLINK's HMM for most relationships. However, Z1estimates were zero for some relationships with an expected coefficient of relatedness ≤0.0625 (Figure 5C; see arrow). In contrast, we obtained low K1 values (Figure 5A; see arrow). Both our K2 and PLINK's Z2 accurately estimated percent IBD2 shared for siblings around the expected value of 0.25 (Figure 5B, 5D).

**Table 2:** IBD estimates in annotated relationships.

IID1	IID2	Expected r	IBS2*_ratio	K0	K1	K2	Z0	Z1	Z2
7	9	0.5	0.940	0.211	0.520	0.269	0.220	0.562	0.219
2	8	0.5	0.938	0.245	0.509	0.246	0.225	0.560	0.216
10	11	0.5	0.999	0.000	1.000	0.000	0.000	1.000	0.000
6	8	0.5	0.999	0.000	1.000	0.000	0.000	1.000	0.000
1	7	0.25	0.836	0.474	0.526	0.000	0.454	0.546	0.000
2	6	0.25	0.826	0.527	0.473	0.000	0.473	0.527	0.000
3	9	0.25	0.857	0.401	0.599	0.000	0.384	0.616	0.000
6	7	0.125	0.759	0.736	0.264	0.000	0.689	0.311	0.000
8	11	0.125	0.738	0.795	0.205	0.000	0.755	0.245	0.000
3	7	0.125	0.763	0.678	0.322	0.000	0.666	0.334	0.000
1	6	0.0625	0.698	0.939	0.061	0.000	0.900	0.100	0.000
1	11	0.0625	0.685	0.953	0.047	0.000	1.000	0.000	0.000
3	12	0.0625	0.697	0.907	0.093	0.000	0.891	0.109	0.000
3	10	0.0625	0.692	0.915	0.085	0.000	1.000	0.000	0.000
3	6	0.03125	0.682	0.956	0.044	0.000	1.000	0.000	0.000
3	4	0.03125	0.673	0.986	0.014	0.000	1.000	0.000	0.000
1	5	0	0.630	0.999	0.001	0.000	1.000	0.000	0.000
2	5	0	0.616	1.000	0.000	0.000	1.000	0.000	0.000

The pedigree has been published [17] and representative relationships are shown. IID1 and IID2, individual identifiers 1 and 2 (see Figure S2); K0, K1, K2 estimates of IBD0, IBD1, IBD2, respectively; Z0, Z1, and Z2, IBD estimates based on PLINK's HMM; Expected r, expected coefficient of relatedness based on annotated relationship.

## Discussion

As the number of genome-wide association studies, SNP datasets, and meta-analyses increase, proper characterization of familial relationships and underlying population structure increases in importance. As an example, Pemberton et al. recently identified a series of unexpected relationships in phase 3 HapMap data [18]. It is expected that the greater the number of individuals in a study, the greater the power to detect variants of small to moderate effect. However, as the size of these studies increases, it is also more likely that related individuals could be introduced or that substantial population substructure develops. Methods to appropriately identify these underlying confounders are key.

In this study we applied visualization and analysis of autosomal IBS and IBD measurements to geographic populations. We introduced a metric based on informative IBS combinations of IBS0 and a subset of IBS2, termed IBS2*, in which AB genotypes are aligned in two individuals at a given chromosomal position. These IBS analyses were complemented by an IBD method that revealed the extent of genome sharing in unannotated related (or known related) pairs of individuals. The main significance of these analyses is that (1) we identified first, second and third degree relationships that were unexpected (for the Human Variation Panel) or consistent with prior annotation (for the validation dataset); (2) we identified population substructure for the geographic groups; (3) we identified individuals who accounted for dramatic variations in the population substructure; (4) knowledge of these individuals may inform future studies that use these datasets; and (5) these combined methods do not require prior information about allele frequencies, ethnic background, or haplotype structure. Our approach is scalable to the study of datasets of any size.

In terms of our IBS approach, Rosenberg [4] applied a closely related approach to an HGDP-CEPH Human Genome Diversity Cell Line Panel and identified close familial relationships from a set of 1,066 samples, while Lee [3] provided a theoretical basis for the method. Some of these ideas have been implemented in PLINK [6] which also provides IBD estimates and has a pairwise concordance test that is also derived from Lee's method. We note that our IBD method called fewer potentially related individuals for whom we could detect no shared alleles on a chromosome-by-chromosome basis, but who had atypical heterozygosity levels with unusually high Z2 estimates and low Z0 estimates. PLINK's HMM results for Z2 estimates for the metachondromatosis pedigree more closely matched expected coefficients (and our K2 estimates), possibly due to a smaller dataset or better annotation.

Allele-sharing methods based on IBS metrics have been widely used [19]. Applications include assessment of population stratification [20], detection of outliers, analysis of pairwise relationships between individuals [21], and linkage analysis [2]. One common approach to visualizing large SNP data sets is principal components analysis (PCA), a technique to reduce the dimensionality of data [22]. Examples include studies of the Han Chinese [23], Europeans [24], [25], [26], [27], Ashkenazi Jews [28], West Africans and African Americans [29], Asians [30], [31], and Indians [32]. PCA allows outlier data points to be identified, and it often results in graphic representation of SNP data that correspond to geographic maps of the populations under study. McVean [33] has shown that the locations of samples in PCA space from genome-wide data can be predicted based on the average coalescent time for pairs of samples. However, the nature of the outliers cannot be assessed (e.g. the occurrence of familial relationships), and it represents an exploratory data analysis approach that is not readily amenable to hypothesis testing of the separation of clusters or of their internal cohesion. Studies based on PCA and the related approach of multidimensional scaling have yielded insights into fundamental population genetics studies such as population stratification or admixture [34], [35], [36] and variation in recombination rate [37].

Plots of mean versus standard deviation of IBS values, such as those by Abecasis and colleagues with Graphical Representation of Relatedness (GRR) [38] and by us [15], are comparable to PCA plots in their ability to represent clusters showing familial relationships, population stratification, or other types of separation. IBS2* plots are even more useful because they provide an objective criterion for defining any pairs of samples as unrelated (IBS2*_ratio value  = 2/3), more related than expected by chance (IBS2*_ratio values >2/3), or less related than expected by chance (IBS2*_ratio values <2/3). The method we introduce is useful for population studies involving even thousands of samples. However, it is also relevant to studies of even a single pedigree. For example, IBS2* plots can be used to confirm reported familial relationships (an essential requirement for successful linkage studies) and to explore the genetic relatedness of individuals who are nominally unrelated but could have more relatedness than expected (e.g. having regions of autozygosity) or less relatedness than expected (e.g. having different ethnic backgrounds).

Apparent genetic relatedness between two individuals could have two independent explanations: shared ancestry (e.g. the two are third cousins) or membership in geographic or ethnic groups that have varying population allele frequencies. The combined IBS and IBD method allowed us to visualize and determine relatedness in the context of either (or both) explanations. The dimension of IBS2*/(IBS0 + IBS2*) reveals relatedness in a manner that is largely independent of population allele frequencies. Our analyses indicated that atypical heterozygosity levels can lead to high IBS2*_ratio values (e.g. 0.70–0.75). Such cases do not necessarily imply familial relatedness and are characterized by two features: (1) relatedness between a given individual and large numbers of others in the population, beyond what is observed in typical pedigrees, and (2) a lack of IBD1 regions on a chromosome-by-chromosome basis, confirming that the individual with atypical heterozygosity is not related to others despite the deviation from a 2/3 IBS2*_ratio value.

While IBS variability can be attributed to familial relatedness and/or to population allele frequencies, another source of variability is the SNP selection process which Clark et al. have shown is subject to ascertainment bias [39]. SNPs detected, annotated, or targeted with a focus on any specific population(s) may not capture the full genetic diversity of other populations. This potential bias provides additional motivation for the introduction of IBS and IBD methodology.

## Methods

### Analysis of NIGMS Human Genetic Cell Repository and other genotype data

The genotypes of 400 individuals from the NIGMS Human Genetic Cell Repository obtained on the Affymetrix Genome-Wide Human SNP Array 6.0 using the Birdseed algorithm were obtained from the Coriell Cell Repositories (accessed June 06, 2008). These collections (n = 100 each) were from AA (HD100AA), CAU (HD100CAU), CHI (HD100CHI; each individual had all four grandparents born in Taiwan, China, or Hong Kong) and MEX (HD100MEX; each individual had either three or four grandparents born in Mexico). In all cases, these individuals were reported to be unrelated and apparently healthy. The data set is available from dbGaP (study accession phs000211.v1.p1). Only autosomal data were used for analysis.

For a validation dataset, we obtained SNP genotype data from a published study that included 12 individuals of ‘known’ relationship [17]. The expected coefficients of relatedness ranged from 1/2 (parent-child and sibling) to 1/32 (first cousins that were twice-removed) and zero (unrelated). There were 66 pairwise comparisons involving all individuals in the pedigree.

### Measurement of IBS

We measured autosomal IBS values using the freely available, cross-platform SNPduo++ software (v1.02) which measures IBS2* from pairwise relationships as well as measurements of IBS0, IBS1, and IBS2 [15] (available for download [40]). The output was imported into Partek Genomics Suite (GS) software (Partek Inc., St. Louis, MO) for visualization and analysis. We also implemented the measurements of IBS and IBS2* statistics within Partek GS software v6.4 that allows easier import of SNP data, measurement of IBS values, and plotting functions.

### IBS2* model

As suggested by Lee [3], all loci having two A alleles and two B alleles represent informative IBS observations that can distinguish between related and unrelated pairs of individuals. IBS0 is here defined as the total number of observations in which two discordant homozygotes are present (e.g. AA/BB) while IBS2* results when two concordant heterozygotes are compared (i.e. AB/AB) between any pair of individuals. The null hypothesis, which assumes that these are unrelated individuals, is that these two individuals have four unrelated alleles, while the alternative hypothesis is that they do not.

We consider alleles A and B having frequencies *p* and *q*, where *p_i_*+*q_i_* = 1 denotes the allele frequency for the *i*th informative locus, with informative markers *i* = 1, 2, … *m* (for *m*<*n* total genotyped SNPs). Conditional probabilities for concordance under the null hypothesis H_0_ (assuming Hardy-Weinberg equilibrium of alleles at any one locus) are given by Lee as follows [3]:
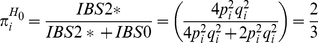
(1)


A notable feature of this approach is that the probabilities are expected to be independent of the population allele frequencies for each SNP and should reduce to 2/3 based on the genome-wide sums of IBS2* and IBS0. The test statistic (*T*_1_), variance, and statistic *Z*_1_ are given in [3]. We estimated probability values based on the *Z*_1_ statistic. Results based on an exact binomial test for p = 2/3 were quantitatively and qualitatively similar (data not shown).

Lee [3] gave conditional concordance probabilities for the two alternative hypotheses in which a ratio greater than 2/3 implied relatedness, and less than 2/3 implied the two individuals are from different populations.

We introduced an IBS2* plot y-axis based on the frequency of informative SNPs as follows: 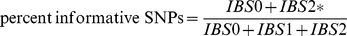
(2)


In contrast to the x-axis (IBS2*_ratio), the allele frequencies which are represented by *p* and *q* do not cancel out. Values for pairwise comparisons displayed on the y-axis (percent informative SNPs) are positively correlated with heterozygosity rates (shown in Figure 1B). The more AB genotypes that are within a population, the more likely to align AB calls when comparing two individuals. This corresponds to a higher IBS2* count which increases the percent informative SNPs (y-axis). We empirically noticed that IBS0 levels have a slight effect on the height of the y-axis but contribute more to the placement on the x-axis (i.e. IBS2*_ratio). This not only applies to the comparison of unrelated individuals, but is true for other relationships such as full siblings and parent-child as well.

### IBD method

We implemented a method for IBD estimation. We present a simplified graphical overview in Figure S3. For each pair of individuals, we removed all concordant homozygous SNPs (i.e. AA/AA, BB/BB) throughout all autosomes resulting in an average of 423,328 SNPs per pairwise comparison. Note that loci having NCs (no calls) in either sample were ignored. We restricted our calculations to windows of 300 SNPs that iteratively overlapped along each chromosome. Within each window, we included IBS0 (e.g. AA/BB) and IBS2* (i.e. AB/AB) SNPs for estimating IBD0/ not IBD0, for which there was an average of 134,880 SNPs with an average genomic length (for the Human Variation Panel) of 6.67 Mb per pairwise comparison. Note that for "not IBD0" states the symbol 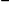 corresponds to "not". For determining whether 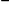
IBD0 states were IBD1 or IBD2, IBS1 (e.g. AA/AB) and IBS2* (i.e. AB/AB) SNPs were used with an average of 377,360 that corresponded to a genomic length of 2.38 Mb per pairwise comparison. We employed a series of window sizes, using 300 as a default size based on empirical observation that it reduced background noise while yielding expected values of IBD. We note that increasing the window size over 600 decreased the estimation of expected IBD values because the boundaries between the different IBD states were not as easily defined. This window size is user-selectable.

For each window, we calculated a likelihood of each IBD state given the observed IBS values. For example, we expected to observe an IBS0 ratio (i.e. IBS0/(IBS2* + IBS0) of 1/3 for IBD0 states, and an IBS0 ratio of 0 for 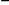
IBD0 states that share 1 or 2 alleles IBD. The samples were assumed to be drawn from the same population with unknown allele frequencies. We express the likelihood of observing a given IBS frequency vector as the marginalization across all IBD states (P(S)), and we define D0, D1, D2, as IBD0, IBD1, IBD2 respectively. 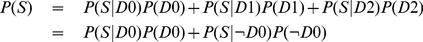
(3)

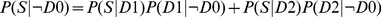
(4)We assume prior IBD probabilities of P(D0) = P(D1) = P(D2) = 1/3, P(D1 | 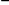 D0) = P(D2 | 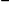
D0) = 1/2. We note that any prior could be used within the algorithm, but without any reason to believe there is a specific relationship, the non-informative prior is a simple choice, and each IBD state is equally as likely to occur.

An observation error rate is also incorporated into the likelihood model. This is fixed for all of the analyses. The probabilities used to compute likelihoods given an IBS call at a SNP for each IBD state assignment are in Table 3 for SNP specific values *p* and *q*.

**Table 3:** IBS probabilities given IBD state.

IBS	IBD0	IBD1	IBD2
0	2p^2^q^2^	0	0
1	4p^3^q+4pq^3^	2pq	0
IBS2*	4p^2^q^2^	(1/2) - pq	2pq

IBS probabilities were derived from Hardy-Weinberg statistics (derived from *p* ^2^+2*pq*+*q* ^2^ = 1). For example, the probability of an IBS0 call in a region of IBD1 is 0.

To estimate P(S | D0), we limit our likelihood calculations to only the IBS0 and IBS2* calls. The observed frequencies of these two IBS states are independent of allele frequency at each SNP when conditioned on an observed IBS0 or IBS2 call, with P(observed S0 | D0, observed S0 and S2)  = 1/3.

We use the following distribution assumptions to create the likelihood of observing the IBS0 frequency. The error (set by default at 0.01) reflects the genotyping error.
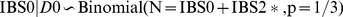
(5)

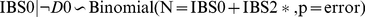
(6)


From this, we have P(S | D0) and P(S | 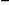
D0), and we compute posterior IBD0 probability of the region.
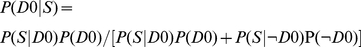
(7)

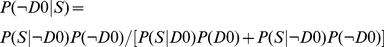
(8)


We use this estimate of P(D0 | S) as our estimated IBD0 probability. The next step is to divide P(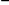
D0 | S) into estimates of P(D1 | S) and P(D2 | S). We use the following distribution assumption to approximate the expected frequency of IBS1 calls (i.e. IBS1/[IBS1 + IBS2*]). The parameter *c* is discussed below. The error rate is set at 0.01.
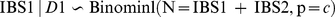
(9)

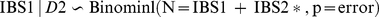
(10)


Using these distributions, we can provide estimates for P(S | D1) and P(S | D2). Since all the three terms of P(S) above are estimated after specifying a prior probability, we can compute the three IBD probabilities, P(D0 | S), P(D1 | S), and P(D2 | S) using Bayes' rule.

We estimate the K coefficients using the estimates of P(D0 | S), P(D1 | S), P(D2 | S) for each window, w, spanning genome length l_w_. P_w_ denotes a probability value across a given window.
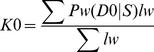
(11)

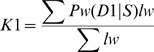
(12)

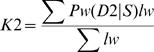
(13)


### Estimation of parameter *c*

The windowed approach used observed IBS to identify regions that were IBD0 or 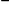
IBD0. In estimating regions of IBD0 or 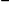
IBD0 the allele frequencies cancelled out (similar to equation [1]). A further step of our algorithm is to distinguish the set of regions that are IBD1 and IBD2 (i.e. the 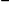
IBD0 regions). It is necessary to account for allele frequencies in these regions. We present a justification for the *c* parameter (equation 9) which is used for distinguishing IBD1 and IBD2 for regions that are 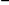
IBD0. For each SNP, the proportion of observing an IBS1 event given that an IBS1 or IBS2* event was observed in a region of IBD1 can be defined as a function, *f*, taking the allele frequency *p*, as defined in Table 3.
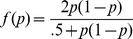
(14)


We can integrate over the allele probability function, P(*p*), to calculate an expected *f*(*p*).
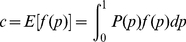
(15)


We can specify P(*p*) to represent any prior belief about the allele frequency of those SNPs that have observed variation. We used a one-step empirical Bayes' estimate of P(*p*) to suggest a practical value.

Assume that an event O occurs when a SNP has observed variation in the two comparison samples.
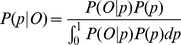
(16)


We define P(O | *p*) in a region of IBD1 as P(O | *p*) = 5+*p* (1-*p*). This is the complement of the chance to observe identical homozygous genotypes in a region of IBD1.

An initial estimate of P(*p*) is specified as a uniform prior over *p*. This initial uniform prior is used to compute a posterior P(*p* | O), which is then used as the empirical prior to compute P_e_(*p* | O).

Using P_e_ (*p* | O) in place of P(*p*) in the calculation of *c* above results in the expectation *c* = .518. The value of *c* should be chosen greater than the error and less than or equal to than the maximum allowable value of 2/3. The assumed binomial model considers all SNPs to be drawn with the same allele frequency which is not accurate, as the binomial parameter *p* varies across SNPs as a function of allele frequency. The binomial model described above was chosen for its simplicity and the experimental insensitivity to choice of *c* across a wide range of reasonable values. Future work may use the beta binomial distribution to better account for the distribution of allele frequencies. We tested a range of *c* values and observed consistent results for K1 and K2 estimates given *c*≥0.25 (data not shown).

The IBD algorithm (called kcoeff) is available as an executable at the authors' website [40], as well as the source code.

### PLINK's HMM analysis

For the Human Variation Panel, we applied PLINK's HMM, given by the "--genome" option, to each group independently (e.g. 100 Mex individuals) because a homogeneous population is recommended [6], [16]. Each group of 100 had the following quality control measures: (1) individuals with ≤98% genotype call rate were removed; (2) SNPs with ≤99% genotype call rate were removed; (3) SNPs with a failure of Hardy-Weinberg equilibrium with a p≤0.0001 were removed; (4) SNPs with a minor-allele frequency (MAF) ≤0.01 were removed. Results using these quality control criteria are shown in Figure 3C, 3D and Figure 4C, 4D. We also ran PLINK's HMM without removing individuals that had ≤98% genotype call rate as shown in Figure 3E, 3F. We summarized the effects of these quality control measures for each of the four Human Variation Panel populations (Table S2).

PLINK's HMM analysis for the validation dataset was run with incomplete pedigree information since data for both parents, which must be specified in a tped file, were not available. Only one trio was specified. We analyzed the 12 family members' data in PLINK using the same quality control measures as above, and no samples were excluded based on low genotyping rate. 7 SNPs were removed due to ≤99% genotype call rate, while 87,234 were removed due to MAF ≤0.01 leaving 450,924 SNPs for analysis.

## Supporting Information

Figure S1 Reconstruction of pedigrees. Pedigrees inferred from IBS and IBD sharing are shown for individuals from CAU, CHI, and MEX populations. Abbreviations: K1, estimate of IBD1 using our method; x, IBS2*_ratio value. For panel B, the relationship of NA17626 to two males (NA17655 and NA17656) is via a father for whom SNP data were unavailable; these relationships are indicated with a dashed line.
(PDF)

Figure S2 Pedigree for validation dataset. Numbers correspond to individuals listed in Table 2, and relationships are plotted in Figure 5. Numbered individuals were those genotyped in pedigree 1 from [17].
(PDF)

Figure S3 Overview of the IBD method. Upper panels: representative IBS observations between two related individuals across a typical chromosomal segment (x-axis). Each data point corresponds to a single locus for which IBS0, IBS1, or IBS2 is observed and plotted (y-axis). We delineate four regions using an iterative approach (typically using 300 informative SNPs per window). In Region 1, having IBS0, we detect IBS0 and infer an IBD0 region. In Region 2, lacking IBS0 calls, we define the locus as “not IBD0.” We infer the IBD1 versus IBD2 state depending on the IBS1 calls (based on allele frequency estimator *c*). Region 3, having observed IBS1 calls, is inferred to be IBD1. Region 4, lacking observed IBS1 calls, is inferred to be IBD2. K0, K1, and K2 estimates are defined in the figure and correspond to Cotterman coefficients of relatedness k0, k1, and k2, respectively.
(PDF)

Table S1 Samples with low genotype rate. Genotype rates for individuals that were removed from PLINK HMM analysis due to low genotype call rate (≤0.980).
(TXT)

Table S2 Quality control information for each ethnic group. Quality control was performed using PLINK to remove individuals with ≤98% genotype call rate, SNPs that failed a HWE (p <0.0001), SNPs that had ≤99% genotype call rate, and SNPS with a MAF ≤0.01. The first MEX, CHI, CAU, and AA columns represent the quality control measures in which individuals with low genotyping rates were removed. The second MEX, CHI, CAU, and AA columns represent the quality control measures in which individuals with low genotyping rates were not removed.
(TXT)
