# Supplementary material for: Inference of Relationships in Population Data Using Identity-by-Descent and Identity-by-State
Source: PLoS Genet. 2011 Sep 22;7(9):e1002287. doi: 10.1371/journal.pgen.1002287 (PMC3178600; doi:10.1371/journal.pgen.1002287)
Supplement: Figure S1 — Reconstruction of pedigrees. Pedigrees inferred from IBS and IBD sharing are shown for individuals from CAU, CHI, and MEX populations. Abbreviations: K1, estimate of IBD1 using our method; x, IBS2*_ratio value. For panel B, the relationship of NA17626 to two males (NA17655 and NA17656) is via a father for whom SNP data were unavailable; these relationships are indicated with a dashed line. (PDF) [file pgen.1002287.s001.pdf]

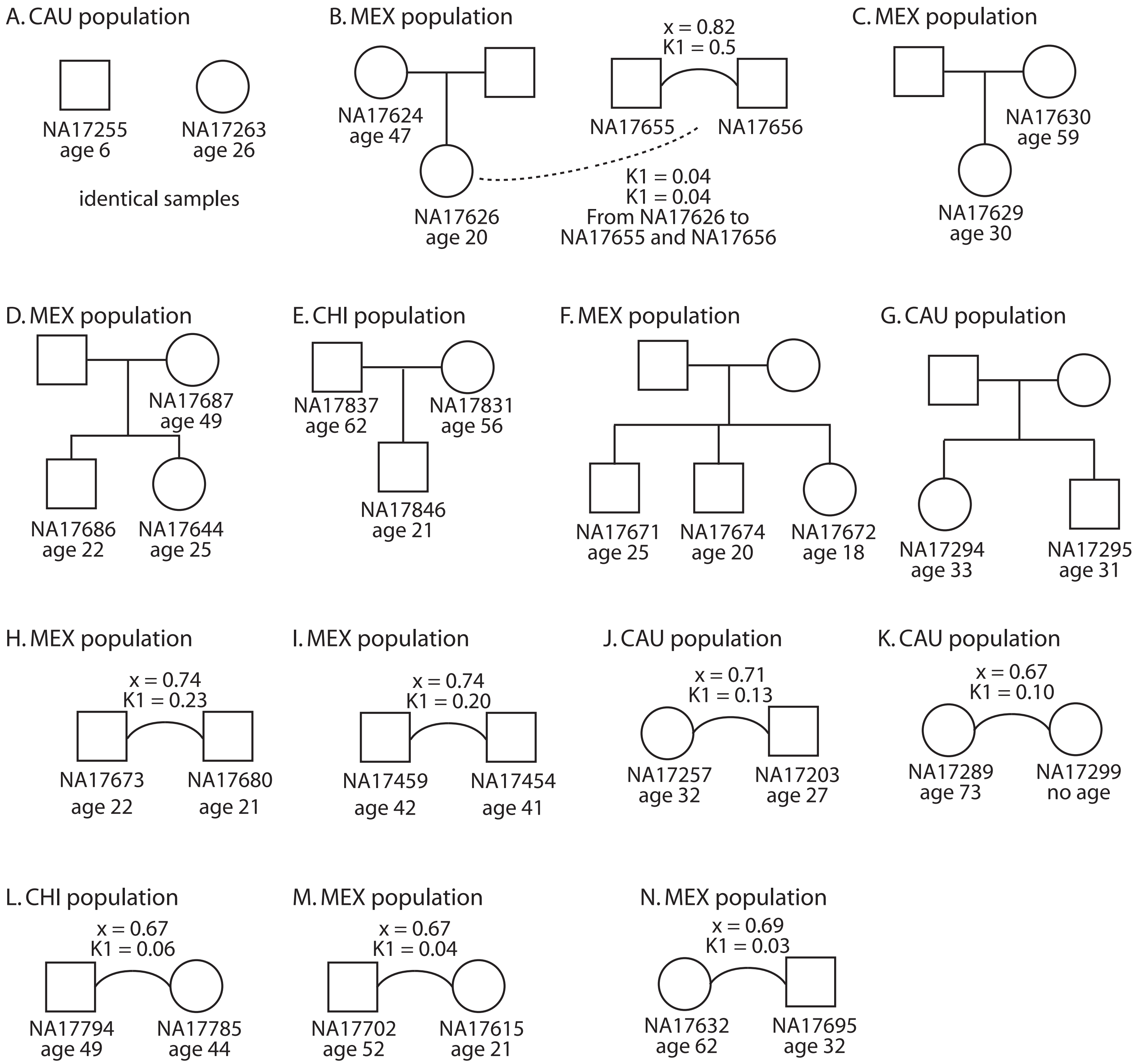
A. CAU population
NA17255
age 6
NA17263
age 26
identical samples
B. MEX population
NA17624
age 47
NA17626
age 20
x = 0.82
K1 = 0.5
NA17655
NA17656
K1 = 0.04
K1 = 0.04
From NA17626 to
NA17655 and NA17656
C. MEX population
NA17630
age 59
NA17629
age 30
D. MEX population
NA17687
age 49
NA17686
age 22
NA17644
age 25
E. CHI population
NA17837
age 62
NA17831
age 56
NA17846
age 21
F. MEX population
NA17671
age 25
NA17674
age 20
NA17672
age 18
G. CAU population
NA17294
age 33
NA17295
age 31
H. MEX population
x = 0.74
K1 = 0.23
NA17673
age 22
NA17680
age 21
I. MEX population
x = 0.74
K1 = 0.20
NA17459
age 42
NA17454
age 41
J. CAU population
x = 0.71
K1 = 0.13
NA17257
age 32
NA17203
age 27
K. CAU population
x = 0.67
K1 = 0.10
NA17289
age 73
NA17299
no age
L. CHI population
x = 0.67
K1 = 0.06
NA17794
age 49
NA17785
age 44
M. MEX population
x = 0.67
K1 = 0.04
NA17702
age 52
NA17615
age 21
N. MEX population
x = 0.69
K1 = 0.03
NA17632
age 62
NA17695
age 32
