# Supplementary figures and images for: Inference of Relationships in Population Data Using Identity-by-Descent and Identity-by-State
Source: PLoS Genet. 2011 Sep 22;7(9):e1002287. doi: 10.1371/journal.pgen.1002287 (PMC3178600; doi:10.1371/journal.pgen.1002287)

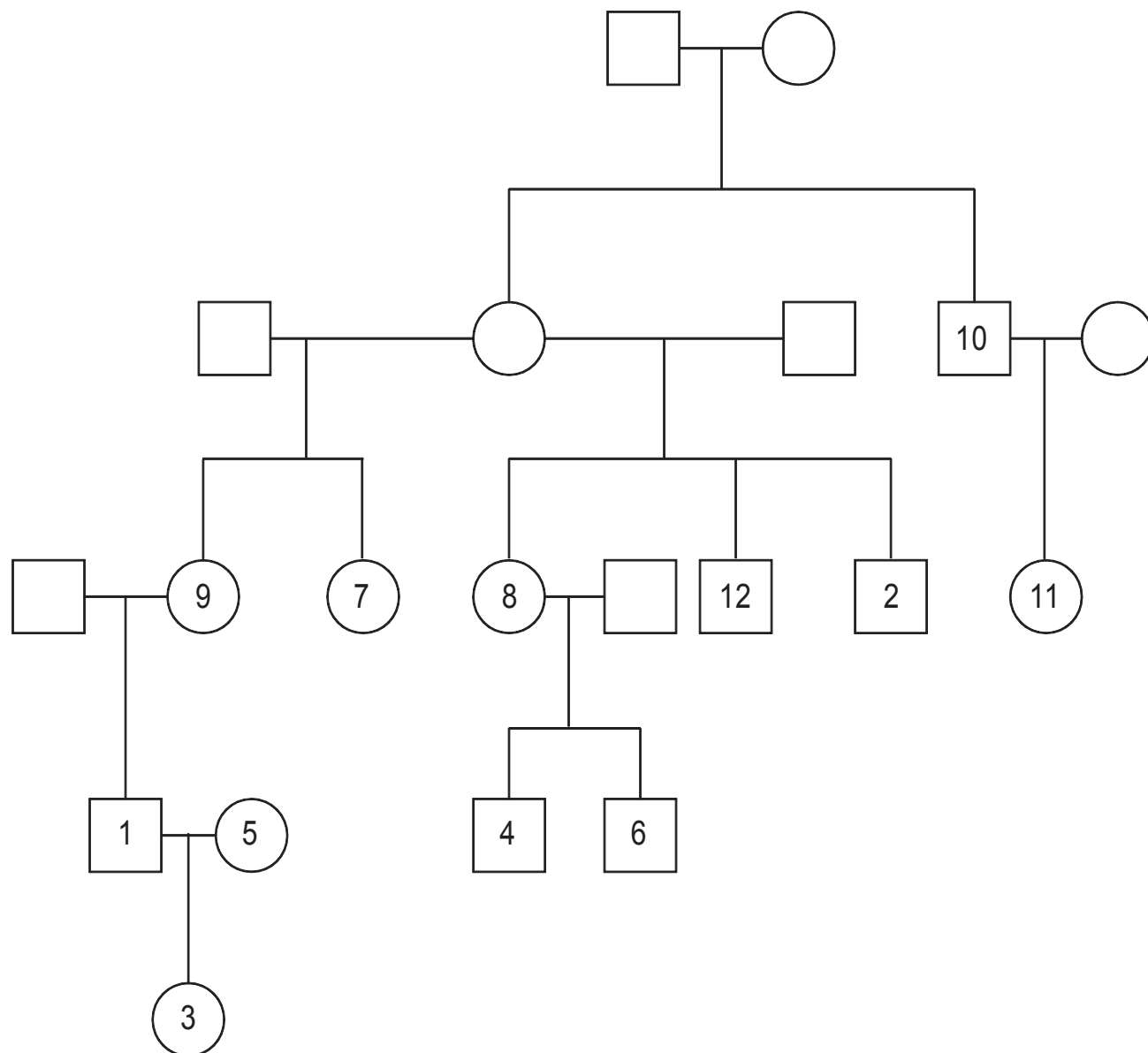

Supplement: Figure S2 — Pedigree for validation dataset. Numbers correspond to individuals listed in Table 2, and relationships are plotted in Figure 5. Numbered individuals were those genotyped in pedigree 1 from [17]. (PDF) [file pgen.1002287.s002.pdf]
