# Supplementary material for: Inference of Relationships in Population Data Using Identity-by-Descent and Identity-by-State
Source: PLoS Genet. 2011 Sep 22;7(9):e1002287. doi: 10.1371/journal.pgen.1002287 (PMC3178600; doi:10.1371/journal.pgen.1002287)
Supplement: Figure S3 — Overview of the IBD method. Upper panels: representative IBS observations between two related individuals across a typical chromosomal segment (x-axis). Each data point corresponds to a single locus for which IBS0, IBS1, or IBS2 is observed and plotted (y-axis). We delineate four regions using an iterative approach (typically using 300 informative SNPs per window). In Region 1, having IBS0, we detect IBS0 and infer an IBD0 region. In Region 2, lacking IBS0 calls, we define the locus as “not IBD0.” We infer the IBD1 versus IBD2 state depending on the IBS1 calls (based on allele frequency estimator c). Region 3, having observed IBS1 calls, is inferred to be IBD1. Region 4, lacking observed IBS1 calls, is inferred to be IBD2. K0, K1, and K2 estimates are defined in the figure and correspond to Cotterman coefficients of relatedness k0, k1, and k2, respectively. (PDF) [file pgen.1002287.s003.pdf]

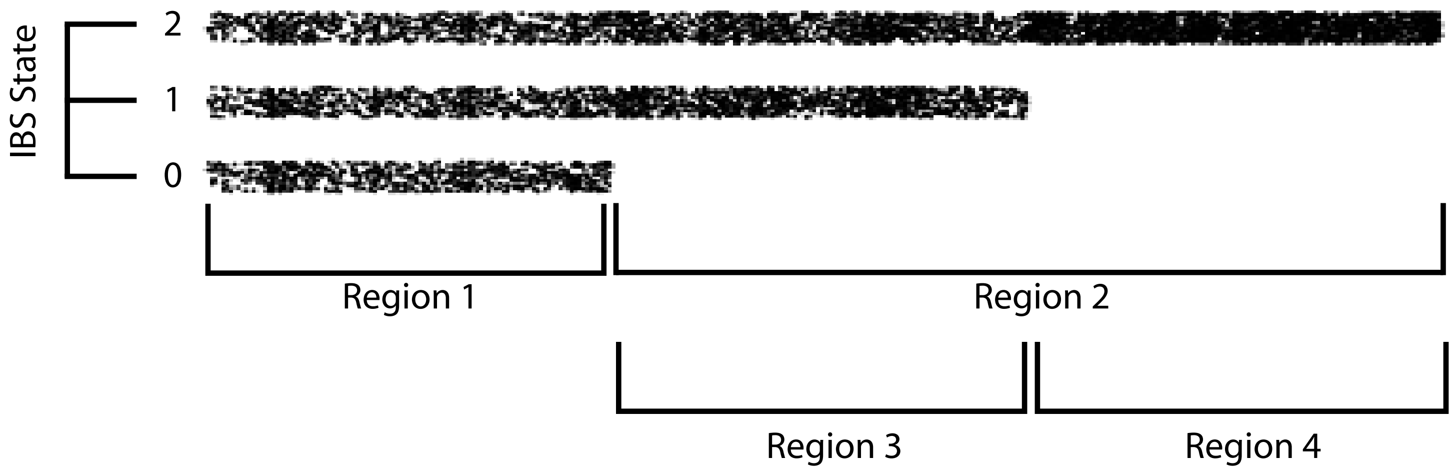

$$\text{Region 1} = \frac{\text{IBS0}}{\text{IBS0} + \text{IBS2*}} = \frac{2p^2q^2}{2p^2q^2 + 4p^2q^2} = \frac{1}{3} = \text{inferred IBD0}$$

$$\text{Region 2} = \frac{\text{IBS0}}{\text{IBS0} + \text{IBS2*}} = 0 = \text{inferred ``not'' IBD0}$$

$$\text{Region 3} = \text{``not'' IBD0} = \frac{\text{IBS1}}{\text{IBS1} + \text{IBS2*}} = c \text{ (see Methods)} = \text{inferred IBD1}$$

$$\text{Region 4} = \text{``not'' IBD0} = \frac{\text{IBS1}}{\text{IBS1} + \text{IBS2*}} = 0 = \text{inferred IBD2}$$

K0 = sum of regions inferred to be IBD0 / total genome length

K1 = sum of regions inferred to be IBD1 / total genome length

K2 = sum of regions inferred to be IBD2 / total genome length
